# Influence of Public Health Services on the Goal of Ending Tuberculosis: Evidence From Panel Data in China

**DOI:** 10.3389/fpubh.2022.826800

**Published:** 2022-03-04

**Authors:** Yang Chen, Qingyu Zhou, Xinmei Yang, Peiwu Shi, Qunhong Shen, Zhaoyang Zhang, Zheng Chen, Chuan Pu, Lingzhong Xu, Zhi Hu, Anning Ma, Zhaohui Gong, Tianqiang Xu, Panshi Wang, Hua Wang, Chao Hao, Chengyue Li, Mo Hao

**Affiliations:** ^1^Research Institute of Health Development Strategies, Fudan University, Shanghai, China; ^2^Collaborative Innovation Center of Social Risks Governance in Health, Fudan University, Shanghai, China; ^3^Department of Health Policy and Management, School of Public Health, Fudan University, Shanghai, China; ^4^Zhejiang Academy of Medical Sciences, Hangzhou, China; ^5^School of Public Policy and Management, Tsinghua University, Beijing, China; ^6^Project Supervision Center of National Health Commission of the People's Republic of China, Beijing, China; ^7^Department of Grassroots Public Health Management Group, Public Health Management Branch of Chinese Preventive Medicine Association, Shanghai, China; ^8^School of Public Health and Management, Chongqing Medical University, Chongqing, China; ^9^School of Public Health, Shandong University, Jinan, China; ^10^School of Health Service Management, Anhui Medical University, Hefei, China; ^11^School of Management, Weifang Medical University, Weifang, China; ^12^Committee on Medicine and Health of Central Committee of China Zhi Gong Party, Beijing, China; ^13^Institute of Inspection and Supervision, Shanghai Municipal Health Commission, Shanghai, China; ^14^Shanghai Municipal Health Commission, Shanghai, China; ^15^Jiangsu Preventive Medicine Association, Nanjing, China; ^16^Changzhou Center for Disease Control and Prevention, Changzhou, China

**Keywords:** tuberculosis incidence, public health services, multisector participation, services assessment, China

## Abstract

**Background:**

The World Health Organization has proposed an initiative to “end tuberculosis (TB).” Unfortunately, TB continues to endanger the health of people worldwide. We investigated the impact of public health services (PHS) in China on TB incidence. In this way, we provided policy ideas for preventing the TB epidemic.

**Methods:**

We used the “New Public Management Theory” to develop two indicators to quantify policy documents: multisector participation (MP) and the Assessable Public Health Service Coverage Rate (ASCR). The panel data from 31 provinces in Chinese mainland were collected from 2005 to 2019 based on 1,129 policy documents and the China Statistical Yearbook. A fixed-effect model was used to determine the impact of MP and the ASCR on TB incidence.

**Results:**

From 2005 to 2019, the average MP increased from 89.25 to 97.70%, and the average ASCR increased from 53.97 to 78.40% in Chinese mainland. However, the development of ASCR between regions was not balanced, and the average level in the western region was lower than that in the eastern coastal provinces. With an increase in MP and the ASCR, the TB incidence had been decreasing gradually in recent years. The panel analysis results showed that MP (β = −0.76, *p* < 0.05). and ASCR (β = −0.40, *p* < 0.01) had a negative effect on TB incidence, respectively. Even if the control variables were added, the negative effects of MP (β = −0.86, *p* < 0.05) and ASCR (β = −0.35, *p* < 0.01) were still statistically significant.

**Conclusions:**

Promoting the participation of multiple departments, as well as emphasizing the quality of PHS delivery, are important ways to alleviate the TB epidemic. The settings of evaluation indices for PHS provision should be strengthened in the future.

## Background

Tuberculosis (TB) is one of the top-10 leading causes of death worldwide, and has become the leading infectious-disease killer in humans (1). The widespread prevalence of TB (especially in low-income countries) has imposed a significant economic burden upon society (2).

The World Health Organization (WHO) proposed a program to eradicate TB by 2035, implying that the incidence rate and mortality rate of TB would be reduced by 90 and 95%, respectively, by 2035 compared with those in 2015 (3). However, about 10 million people worldwide were suffering from TB in 2019, and the TB incidence in China was still >58 per 100,000 people (1).

A growing body of research has indicated that providing adequate, high-quality, and accessible public health services (PHS) for TB would help to alleviate the TB epidemic (4). For example, introduction of the rapid molecular test can reduce the disequilibrium of TB prevalence among regions significantly (5). The TB prevalence rate in half of regions in China decreased by 36% in 2000 since implementation of the Directly Observed Treatment, Short-Course strategy in 1990 (6). In addition, the promotion of health education and health awareness can reduce the delay in the TB diagnosis (7). Improving the utilization rate of PHS for TB could reduce the incidence and mortality in high-risk populations (8). In South Africa, discontinuation of TB treatment services for children was the main cause of the continuous deterioration of TB mortality (9). The sustainable development goals of the WHO and World Bank defined “universal health coverage” as universal access to the health services they need (10), which included basic prevention, treatment, and care interventions. The WHO also recommended that countries should take systematic and robust action to increase access to TB-prevention services and other preventive projects (11).

In China, public sectors, including government departments, hospitals and public health institutions, are the most significant factors in the management and provision of public services. According to the “New Public Management” theory, public sectors driven by customer orientation can provide high-quality public services (12). This strategy requires the public sectors to participate in the provision of public services required by customers (13), and paying attention to the efficiency and quality of provision of public services (14). PHS for TB also need to emphasize the active participation of departments and service quality. More departments involved in service delivery for the prevention and control of TB, taking responsibility together with the health departments. Meanwhile, PHS can be launched in different populations and regions, which greatly improves the efficiency of prevention and control of TB. Furthermore, the requirement for PHS quality can save time and cost while achieving effective use of resources, which helps in the prevention and control of TB.

Several studies have examined the multisectoral participation and service quality of PHS for TB. When it is not possible to provide adequate PHS to the public, it is essential to develop policies to strengthen multisectoral involvement and promote cross-sectoral collaboration (15, 16). Oluwasanu and colleagues evaluated the provision of PHS for TB using the assessment tool for service availability of the WHO through semi-structured interviews. They revealed the shortage of quality PHS for TB from qualitative data (17). Another important approach to analyzing PHS quality is assessment of service accessibility. One survey found that the coverage rate of the management service for TB patients in China was low, and needed to be improved further (18). In addition, although providing vaccination is an effective measure for TB control, there are difficulties in implementing the vaccination strategy fully. Thus, monitoring and screening need to be strengthened in the meantime (19). Another study analyzed the quality of PHS for TB through the satisfaction of service objects (20). However, most of those studies relied on surveys or interviews with the general population to analyze the multisectoral participation and quality of service delivery of PHS for TB. We believed that it was necessary to conduct further research on the status of PHS for TB based on policy documents.

We examined the impact of PHS for the prevention and control of TB based on the perspective of policy documents from two aspects, including multisectoral participation and service quality. In the context of sustainable development, this study will help to identify the shortcomings in the PHS for TB in China and provide a reference for subsequent strategies of strengthening the prevention and control of TB, in order to achieve the ultimate goal of eliminating TB.

## Methods

### Study Design

Analysis of policy documents was used to ascertain the evolution of PHS for TB in 31 provinces in Chinese mainland from 2005 to 2019. A fixed-effect model was employed to analyze the impact of PHS on the effectiveness of the prevention and control of TB based on the panel data of these 31 provinces.

The starting point for policies included in this analysis was set to 2005. First, in 2005, China achieved the modern TB control strategy proposed by the WHO and upgraded to the Stop TB strategy in accordance with international efforts (20). Second, almost all provinces issued 5-year plans for the prevention and control of TB in 2005 under the national demand that all regions step-up construction of systems for the prevention and control of TB. Third, TB surveillance system in China was set up in 2005 (21), which ensured the availability and accuracy of the data.

The incidence rate was selected as an indicator to measure the effectiveness of PHS for TB, which was widely used to measure the effectiveness of the prevention and control of infectious diseases in China and abroad (1, 22). Moreover, TB incidence was monitored in 31 provinces, which ensured the validity of our data.

### Definition of PHS for TB

Based on guide and handbooks issued by the WHO on the prevention and control of TB (23, 24), and the consultation with experts, the PHS for TB were summarized into eight categories (Table 1): health education; risk factors monitoring; behavioral intervention; vaccination; controlling the source of infection; discovery and detection; case finding and reporting; management and follow-up.

**Table 1:** Definition of public health services for tuberculosis.

**Service category**	**Details**
Health education	Conducting health education on prevention knowledge and prevention measures of tuberculosis.
Risk factors monitoring	Carrying out monitoring of population epidemics, host, vectors, and other epidemic factors, and conducting epidemiological investigations.
Behavioral intervention	Conducting interventions for risky behavior.
Vaccination	Supplying national immunization programs and routine vaccinations.
Controlling the source of infection	Tracking and managing patients rapidly, and identifying the contacts of cases who have been infected.
Discovery and detection	Early diagnosis of TB (surveillance initiative, population screening) and systematic screening of contacts and high-risk groups.
Case finding and reporting	Finding and reporting cases, reporting information of infectious diseases, and monitoring TB notifications, prevalence and trends.
Management and follow-up	Supplying case management, case treatment, and follow-up management.

### Indicator Design

We designed two quantitative indicators according to the New Public Management theory, emphasizing departments' participation and service quality.

In the departments' participation, the WHO recommended that countries need collaboration across relevant departments, such as finance and justice, to implement the end TB strategy (3). This requires that a particular department entity assume responsibility for a specific PHS. For example, health commission, public health institutions, hospitals, and primary healthcare providers undertake the PHS of screening, early diagnosis and treatment, education department and schools undertake health education for students, and radio and television administrations undertake health education and promotion to the public. We consider them all to be participating departments. The number of departments responsible for PHS for TB can illustrate the scope of multisectoral involved. So we designed an indicator called “multi-sector participation” (MP) to reflect the degree of multisectoral participation (24), which can be measured by the proportion of departments with responsibilities among the departments that should undertake PHS for TB. This study assumed that a higher MP implied a greater extent of multisectoral engagement.

In the service quality, the establishment of assessment indicators for PHS is a key measure to ensure service implementation, which could guarantee the efficiency and quality of services. The WHO recommends countries use operational indicators such as treatment coverage to facilitate implementation of PHS (3). Therefore, we designed the indicator Assessable Healthcare Service Coverage Rate (ASCR) (25) to indicate the degree of attention to the quality of service delivery, which can be measured by the proportion of service types with evaluable indicators in PHS. We assumed that a high value of the ASCR indicated that PHS could be provided more effectively than a low value of the ASCR.

According to policy documents from the WHO and China (26, 27), at least 21 stakeholders should be involved in PHS for TB: provincial governments; health commissions; public health institutions; hospitals; primary healthcare providers; development and reform commissions; departments of finance; human resources and social security departments; healthcare security administrations; departments of education; departments of civil affairs; departments of agriculture and rural affairs; departments of commerce; departments of transport; departments of justice; administrations for market regulation; entry–exit inspection and quarantine bureaus; public security departments; radio and television administrations; customs; non-governmental organizations.

### Data Collection

The data sources for the two indicators were policy documents published by provincial departments on official Internet websites.

First, we searched all policy documents from 2005 to 2019 on the official Internet websites of provincial-government departments. The titles and contents contained keywords such as “infectious diseases” and “tuberculosis.” The Internet websites visited included those of local governments, the Health Commissions, the Centers for Disease Control and Prevention, and other departments. The inclusion criteria for documents were: (i) publicly released policy documents, including laws, regulations, plans, schemes and standards; (ii) released between 2005 and 2019; (iii) 31 provincial-level documents in the Chinese mainland. The exclusion criteria were: (a) news, reports or other documents which had no administrative validity; (b) documents with an unknown release date. A total of 1,129 documents from 31 provinces were included in the study.

Second, we extracted and encoded the key information required for the two indicators in the documents. After unified training and trial coding, we independently coded the two indicators in the documents (Appendix 1). Taking ASCR as an example, if a category of PHS was mentioned in a document, the PHS category was coded as “1.” Meanwhile, if an evaluation index was also set for the PHS category, the evaluation index for PHS category was coded as “1.” For example, *the Beijing Tuberculosis Control Standard* mentioned that tuberculosis patients are tracked by TB prevention and control institutions within the jurisdiction, and the follow-up rate for tuberculosis patients need to achieve 80%, which means the PHS category of management and follow-up was considered provided with evaluation index, so we coded the PHS category of management and follow-up as “1” and the evaluation index for management and follow-up was coded as “1” too. We attached the original description from the file to the document upon encoding for subsequent verification and correction of the encoding results.

Third, we tested the reliability of the data-collection process using the test–retest reliability method with the intra-group correlation coefficient (ICC). The ICC was found to be 0.997 after retesting by two experienced researchers, and showed a high level of confidence in the data-collection.

The socioeconomic data in the panel data came from the *Chinese Health Statistics Yearbook, Chinese Economic and Social Development Yearbook*, and *Chinese Statistics Yearbook*.

### Estimation Strategy

We constructed the panel data of 31 provinces in Chinses mainland from 2005 to 2019. Then, we analyzed the impact of MP and the ASCR on TB incidence using a fixed-effect model. To improve the accuracy of results, we simultaneously controlled the fixed effect of provinces and years, and established a regression model for the core variables first and then added the control variables to establish a regression model (28). The models were built as follows:


(1) $$\begin{array}{r} I_{it}=\alpha +\beta_{1}{MP}_{it}+\delta_{t}+u_{i}+\varepsilon_{it} \end{array}$$

(2) $$\begin{array}{r} I_{it}=\alpha +\beta_{1}{ASCR}_{it}+\delta_{t}+u_{i}+\varepsilon_{it} \end{array}$$

(3) $$\begin{array}{r} I_{it}=\alpha +\beta_{1}{Control}_{it}+\beta_{2}{MP}_{it}+\beta_{3}{ASCR}_{it}+\delta_{t}+u_{i}+\varepsilon_{it} \end{array}$$

In the three models, *I*_*it*_ was the interpreted variable, which represented the annual TB incidence in each province. *MP*_*it*_ and *ASCR*_*it*_ were the two core explanatory variables in our study. *Control*_*it*_ were four control variables, which represented the number of beds in medical institutions (10,000), health workforce per thousand population, the natural log of the population density, and proportion of the rural population. They were selected as control variables because they had been confirmed as influencing factors for TB incidence in China in previous studies (29–31). δ_t_ and *u*_*i*_ represented the year and province fixed effect, respectively. ε_*it*_ was the random error term. α, β_1_, β_2_, β_3_, and β_4_ were the parameters to be estimated.

### Statistical Analysis

Excel™ 2019 (Microsoft, Redmond, WA, USA) was used for extraction of policy documents and database collation. Panel data were analyzed using Stata 14.0 (Stata, College Station, TX, USA). *P* < 0.05 was considered significant.

To analyze the disparity on indicators across regions, ASCR and incidence were presented as means by region [eastern, central, western, and northeastern (32)] for 2019.

## Results

### Descriptive Analysis of MP and the ASCR

#### Trends of Average MP and ASCR in 31 Provinces

**Figure 1:**
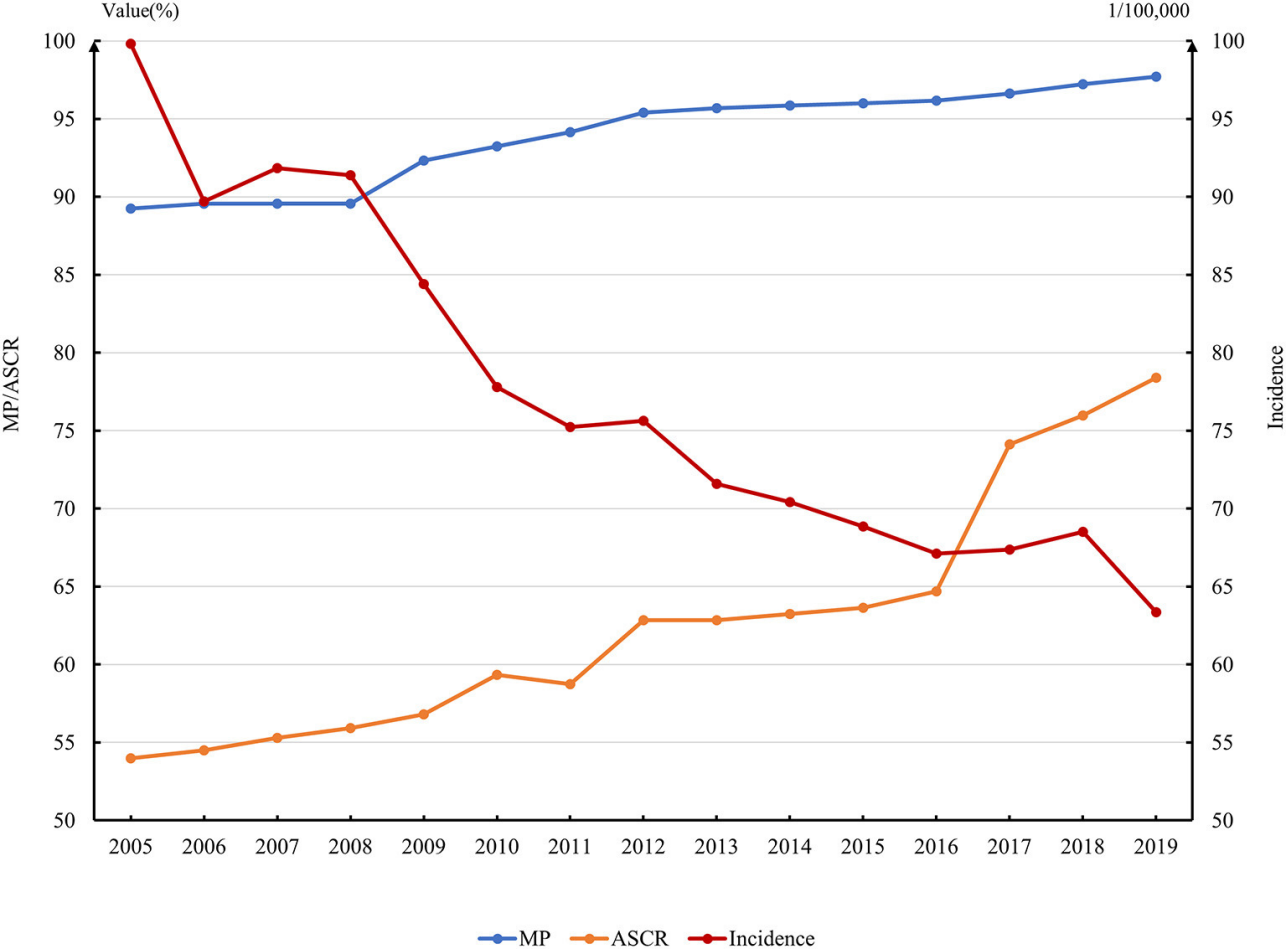
Annual variation of MP, ASCR, and Incidence in 31 provinces in China.

Figure 1 shows steady growth trends of the average MP and average ASCR over the 15 years in 31 provinces. In particular, the ASCR had increased significantly in the past decade. From 2005 to 2019, the ASCR increased from 53.97 to 78.40%, which indicated that the provinces had established TB-control programs actively and strengthened evaluation of PHS based on WHO and national initiatives. In particular, due to the nationwide deployment of the Healthy China Strategy, the growth after 2016 was extremely remarkable. The sectors involved in PHS for TB in 31 provinces already in high positions in 2005 and had covered all 21 sectors gradually, with an average MP of 97.70% in 2019.

#### The ASCR of 31 Provinces in 2019

The availability of PHS was generally high in all regions. Only one province does not require the PHS of discovery and detection in 2019, while the other seven categories of PHSs are required in all 31 provinces. More specifically, eight provinces had set assessment indicators for behavioral intervention, 17 provinces for risk factors monitoring, 20 provinces for behavioral intervention, 28 provinces for health education and discovery and detection, 31 provinces for vaccination, case finding and reporting and management and follow-up. Although the average level of ASCR reached nearly 80% in 2019, there were prominent dissimilarity among regions to regions (Table 2). In general, the ASCR had a high proportion in eastern region. Beijing, Shanghai, Zhejiang, Jiangsu, and Hainan had set evaluation indices for all PHS categories, which were accompanied by the highest ASCR. Conversely, the setting level of PHS evaluation indicators in western provinces was relatively low, even <50% in some areas.

**Table 2:** Disparity analysis of ASCR and incidence by region in 2019.

**Region**	**ASCR (%)**		**Incidence (1/100,000)**	
	**Mean**	**F**	**Mean**	**F**
East	96.25	9.195^***^	40.66	3.260^**^
Central	79.17		59.70	
West	78.13		74.40	
Northeast	66.67		51.94	

** *p < 0.05*,

*** *p < 0.01*.

### Descriptive Analysis of TB Incidence

#### Trends of Average TB Incidence in 31 Provinces

During the past 15 years, the TB incidence had decreased from nearly 100 per 100,000 to 63.36 per 100,000 (Figure 1), with a marked decline observed after 2008. During 2013 to 2018, it showed a slower downward trend, from 71.79/100,000 to 67.38/100,000.

#### TB Incidence in 31 Provinces in 2019

The TB incidence in the western region was, in general, higher than that in the eastern region (Table 2), in contrast to the distribution of the ASCR. Especially in Beijing, Shanghai, and other eastern coastal provinces with high ASCR, the TB incidence was low (32.22 per 100,000 in Beijing and 26.72 per 100,000 in Shanghai).

### Model Results of Panel Regression

Results of panel data analysis with the incidence as a dependent variable are presented in Table 3. Model 1 included only MP, which indicated a negative impact on morbidity (β = −0.76, *p* < 0.05). Model 2 revealed that the ASCR exhibited a significant negative correlation with the TB incidence (β = −0.40, *p* < 0.01). When controlled for all variables, we found that the MP and the ASCR still significantly associated with TB incidence. Specifically, the corresponding TB incidence decreased by 0.85 units for 1 percentage point increase in the MP, and by 0.35 units for each 1% increase in ASCR.

**Table 3:** The relationship between MP, ASCR, and the incidence.

**Variables**	**Model 1**	**Model 2**	**Model 3**
MP^a^	−0.76^**^		−0.85^**^
	(0.34)		(0.31)
ASCR^b^		−0.40^***^	−0.35^***^
		(0.07)	(0.07)
HWPT^c^			−2.30^**^
			(1.06)
NBMI^d^			−0.99^***^
			(0.18)
PD^e^			3.56^**^
			(1.57)
PRP^f^			0.30^***^
			(0.07)
Constant	160.15^***^	126.93^***^	180.55^***^
	(31.09)	(8.05)	(30.16)
Obs	465	465	465
R^b^	0.86	0.88	0.88
F	62.71	66.78	70.10

a *MP, The multi-sector participation*;

b *ASCR, the assessable public health service coverage rate*;

c *HWPT, the health workforce per thousand population*;

d *NBMI, the number of beds in medical institutions*;

e *PD, the (natural log of) population density*;

f *PRP, the proportion of rural population*.

*Standard errors in parentheses*.

** *p < 0.05*,

*** *p < 0.01*.

## Discussion

This was the first study to analyze the impact of PHS on the prevention and control of TB through policy documents and panel data from 31 provinces in the Chinese mainland. We provided the basis for future policy development aimed to strengthen the effectiveness of the prevention and control of TB in developing countries such as China.

We revealed that, if the extent of multisectoral participation of PHS increased, the effectiveness of prevention-and-control work would be better demonstrated. Scholars have proposed that multiple departments should strengthen their participation to ulteriorly restrain the TB epidemic (15, 33). To achieve the goal of promoting health, other sectors besides the health administration and institutions should also be involved in the action (34). Socioeconomic development can reduce the prevalence of TB (35). And the increasingly refined social division of labor makes it inevitable that policy-making departments, construction departments, and public security departments will be involved in the prevention and control of TB (36). Unfortunately, because the pressure of prevention and follow-up of TB is concentrated in community health-service centers, the work of prevention and control of TB places a great emphasis on the responsibilities of the department in health systems. In terms of PHS such as health education, services for different groups can be undertaken by different departments. For example, social organizations can participate in publicity for the public. Meanwhile, the publicity and education of students would be undertaken more advantageously by the department of education. On the other hand, multisectoral participation can promote intersectoral collaboration and, thus, increase the efficiency of the prevention and control of TB (37). The discovery and detection of TB patients require considerable human, financial, and equipment investments, and cannot be fully guaranteed in the short-term if relying only on the actions of health administration and institutions (38). On the contrary, if the development and reform commission and the department of financial play their parts fully within their respective responsibilities and form a cooperative mode, the timeliness of input of various resources can be guaranteed and the health institutions can carry out testing services more efficiently.

Our research demonstrated that setting clear evaluation indicators for PHS can help reduce morbidity. This effect can be attributed to three possible reasons. First, establishment of assessment indicators or criteria in action plans and guidelines indicates the government's emphasis, which urges each responsible sector to take steps to address the risk factors for TB (39). Second, quantitative assessment indicators can guide the responsible departments to carry out PHS more specifically (40). For example, Fujian Province issued the *Notice on Examination Methods and Standards for TB Control Project Work*, which sets the examination standards for TB funding as well as the discovery and care of patients. This strategy ensures that departments at provincial, municipal and county levels participate actively in the provision of health services, thereby improving the efficiency of prevention and control of TB significantly. Both the prevalence of patient discovery and prevalence of cure of TB are higher than expected (41). Third, quantitative indicators aid the monitoring the quality of PHS, and strengthening supervision is an effective measure to strengthen PHS for TB (42). Early detection of TB patients and control of the infection source are crucial to stop rapid and contagious infection (43). In conclusion, establishment of evaluable indicators for each category of PHS for TB is essential.

We found that MP and the ASCR increased gradually over the past 15 years. This finding has also been demonstrated in studies indicating that China has gradually launched preventive treatment for high-risk groups of people infected during the incubation period and strengthened infection control continuously in high-risk areas (44). The improvement in PHS for TB is beneficial for three main reasons. First, China attaches great importance to the work of TB prevention and control, and has improved the system gradually. *The National Tuberculosis Control Plan (2001–2010)* (45) promulgated in 2001 marked the beginning of full implementation of the modern TB-control strategy in China. After reform of the medical service system in China in 2009, the services for the prevention and control of TB have improved (46), and various forms of health-promotion activities have been carried out. Even during the epidemic of Coronavirus Disease 2019 in 2020, the National Health Committee of China issued a notice requesting all localities to expand laboratory tests to ensure the normal PHS, including TB prevention and control (47). Simultaneously, provinces issued more detailed strategies and targets according to local conditions (48, 49). Second, governments at all levels have been increasing their financial investments in the prevention and control of TB (50). Third, China actively strengthened cooperation with international organizations while innovating control methods of TB. For example, the China-Gates TB Project in 2012 has improved the utilization rate of TB health services in poor areas (51).

Nevertheless, the ASCR showed unequal distribution between the eastern and western regions of China. This difference in PHS quality between the eastern and western regions has also been documented (52). For example, compared with eastern provinces, residents of some western provinces are not sufficiently informed about TB prevention and treatment (53), and free testing and treatment policies are not widely available (54). For the eastern region, high population density and population mobility lead to higher imported risks of TB transmission (55). Thus, to prevent the imported risks and maintain the existing effect of prevention and control, the eastern government needs to strengthen the service quality continuously. In addition, the healthcare needs of the population are ignored readily in regions with weak economic development (56). This may be another reason for the absence of an evaluation index for PHS in China's relatively undeveloped western region.

### Limitations

Our study had four main limitations. First, the evidence for content analysis was derived primarily from public policy documents, which may have been incomplete. Although we organized the files on Internet websites as comprehensively as possible and set a unified standard for content analysis, the judgment of research members was inevitably subjective. Simultaneously, some undisclosed policy documents may have been omitted, which may have affected the research results. Second, we analyzed PHS using only the indicators of MP and the ASCR, but there are other indicators for measuring PHS. Further studies need to consider other factors, such as resource allocation and service fairness, to further verify the impact of PHS on the prevention and control of TB. Third, this study focused on the impact of multisectoral participation and overall PHSs quality on the incidence of TB. Further investigations are needed to identify which category of PHSs or sectors contributes the most to reducing TB incidence. Finally, in the analysis of the impact of PHS on TB prevention and control, we selected only the incidence rate as the result index. We can use other result indicators, such as mortality, in the future.

## Conclusions

This study was the first attempt to quantify the impact of PHS on the prevention and control of TB through analysis of policy documents. We demonstrated that if multiple departments participate in PHS simultaneously and emphasize the quality and effect of services, PHS can be implemented adequately, which is conducive to the realization of public-health goals. This finding indicates that policymakers need to quantify the quality and effectiveness of PHS when formulating policies for the prevention and control of TB and other diseases. Nevertheless, the ASCR can still be further improved. Provinces need to continue to strengthen the PHS quality by developing quantitative assessment indicators. Simultaneously, further enhancement of the degree of multisectoral participation is necessary to achieve the end TB strategy.

## Data Availability Statement

The raw data supporting the conclusions of this article will be made available by the authors, without undue reservation.

## Author Contributions

MH and CL conceptualized and designed this study. PS, QS, ZZ, ZC, CP, LX, ZH, AM, ZG, TX, PW, HW, and CH developed methodologies for data collection and analysis. YC, QZ, and XY participated in data collection and analysis. YC and QZ participated in writing and editing. All authors contributed to the article and approved the submitted version.

## Funding

This study was funded by Tsien Hsue-shen Urbanology Award of Hangzhou International Urbanology Research Center and Zhejiang Urban Governance Studies Center [Grant Number: 21QXS004], the Three-Year Action Plan of Shanghai Municipality Strengthens Public Health System Construction [Grant Numbers: GWIV-32 and GWV-12], the National Natural Science Foundation of China [Grant Numbers: 72074048 and 71774031], and the Shanghai Foundation for Talents Development [Grant Number: 2020128].

## Conflict of Interest

The authors declare that the research was conducted in the absence of any commercial or financial relationships that could be construed as a potential conflict of interest.

## Publisher's Note

All claims expressed in this article are solely those of the authors and do not necessarily represent those of their affiliated organizations, or those of the publisher, the editors and the reviewers. Any product that may be evaluated in this article, or claim that may be made by its manufacturer, is not guaranteed or endorsed by the publisher.

## Abbreviations

PHS: public health services
TB: tuberculosis
MP: multisector participation
ASCR: Assessable Public Health Service Coverage Rate
HWPT: the health workforce per thousand population
NBMI: the number of beds in medical institutions
PD: the (natural log of) population density
PRP: the proportion of rural population
Obs: observations.
