# Supplementary material for: Influence of Public Health Services on the Goal of Ending Tuberculosis: Evidence From Panel Data in China
Source: Front Public Health. 2022 Mar 4;10:826800. doi: 10.3389/fpubh.2022.826800 (PMC8931334; doi:10.3389/fpubh.2022.826800)
Supplement: Supplementary file 1 [file Data_Sheet_1.DOCX]

**APPENDIX 1**: Coding instruction of 2 indicators

| **Information** | **Description** | **Coding** |
| --- | --- | --- |
| Department classification | Record department names: | 0- Not mentioned;  1- Department/organization participated |
|  | - Provincial government |  |
|  | - Health commission |  |
|  | - Public health institutions |  |
|  | - Hospitals |  |
|  | - Primary healthcare providers |  |
|  | - Development and reform commission |  |
|  | - Department of finance |  |
|  | - Human resources and social security department |  |
|  | - Healthcare security administration |  |
|  | - Department of education |  |
|  | - Department of civil affairs |  |
|  | - Department of agriculture and rural affairs |  |
|  | - Department of commerce |  |
|  | - Department of transport |  |
|  | - Department of justice |  |
|  | - Administration for market regulation |  |
|  | - Entry-exit inspection and quarantine bureau |  |
|  | - Public security department |  |
|  | - Radio and television administration |  |
|  | - Custom |  |
|  | - Non-governmental organizations |  |
| Public health service classification | Record service names: | 0- Not mentioned;  1- Public health service mentioned in the policy documents |
|  | - Health education |  |
|  | - Monitoring risk factors |  |
|  | - Behavioral intervention |  |
|  | - Vaccination |  |
|  | - Controlling source of infection |  |
|  | - Discovery and detection |  |
|  | - Monitoring and reporting |  |
|  | - Management and follow-up |  |
| Assessment indicator | Record services | 0- Not mentioned;  1- Public health service assessment indicators mentioned in the policy documents |
